# Beyond cardiac risk factors: non-cardiovascular comorbidities in sudden cardiac death prediction

**DOI:** 10.3389/fcvm.2026.1728987

**Published:** 2026-01-22

**Authors:** Thien Tan Tri Tai Truyen, Vu Ngoc Anh Pham, Huong-Dung Thi Nguyen

**Affiliations:** 1 Faculty of Medicine, Nam Can Tho University, Can Tho City, Vietnam; 2 Can Tho Central General Hospital, Can Tho City, Vietnam

**Keywords:** non-cardiovascular comorbidities, predictive model, risk predictors, sudden cardiac death, ventricular fibrillation

## Abstract

Sudden cardiac death (SCD) causes 180,000–360,000 annual deaths in the United States, with mortality rates exceeding 90%. Despite advances in resuscitation science, predicting SCD remains challenging due to inconsistent definitions, subtle warning signs, and temporal variability in risk factors. While traditional cardiovascular conditions are well-integrated into risk prediction models, non-cardiovascular comorbidities remain significantly underutilized despite contributing to nearly 40% of SCD cases. This review examines evidence linking various systemic conditions to SCD risk. Neurologic disorders including epilepsy (1.6–5.89-fold increased risk), depression (1.6–2.7-fold), and anxiety (1.6-fold) elevate SCD vulnerability through autonomic dysregulation and medication effects. Respiratory conditions like COPD (1.3–3.6-fold) and obstructive sleep apnea (1.6–2.6-fold) contribute through chronic hypoxemia and inflammation. Hepatic pathology, kidney disease, anemia, and endocrine disorders (particularly diabetes with 1.7–2.4-fold risk) also demonstrate significant associations. Critically, non-cardiovascular comorbidities predict not only SCD occurrence but also initial cardiac rhythm presentation—essential for determining implantable cardioverter-defibrillator candidates, as these devices only benefit shockable rhythms. Conditions like epilepsy, depression, COPD, liver cirrhosis, and chronic kidney disease correlate with predominantly non-shockable presentations. Current prediction models incorporate few non-cardiac conditions, primarily due to historical cardiac-centric approaches, sample size constraints, complex disease interactions, and overfitting concerns. Proposed solutions include multidisciplinary research collaboration, multicenter data pooling, and advanced machine learning techniques to develop more comprehensive and accurate SCD prediction algorithms.

## 1 Introduction

Sudden cardiac death (SCD) is one of the leading causes of death worldwide. Annual incidence rates vary significantly by age: 1–2 cases per 100,000 in individuals under 35–40 years, 50–100 per 100,000 in middle-aged adults, and over 200 per 100,000 in those aged 75 and older (1–4). In the United States, SCD is responsible for 180,000 to over 360,000 deaths each year (5–7). Despite advances in resuscitation science, return of spontaneous circulation and survival to hospital discharge rates, though improving, remain unacceptably low. With a worldwide mortality rate often exceeding 90% of an cardiac arrest event, SCD remains one of the deadliest conditions, highlighting the crucial need to improve our ability to predict and reduce the burden of this condition (1, 8).

**Figure 1:**
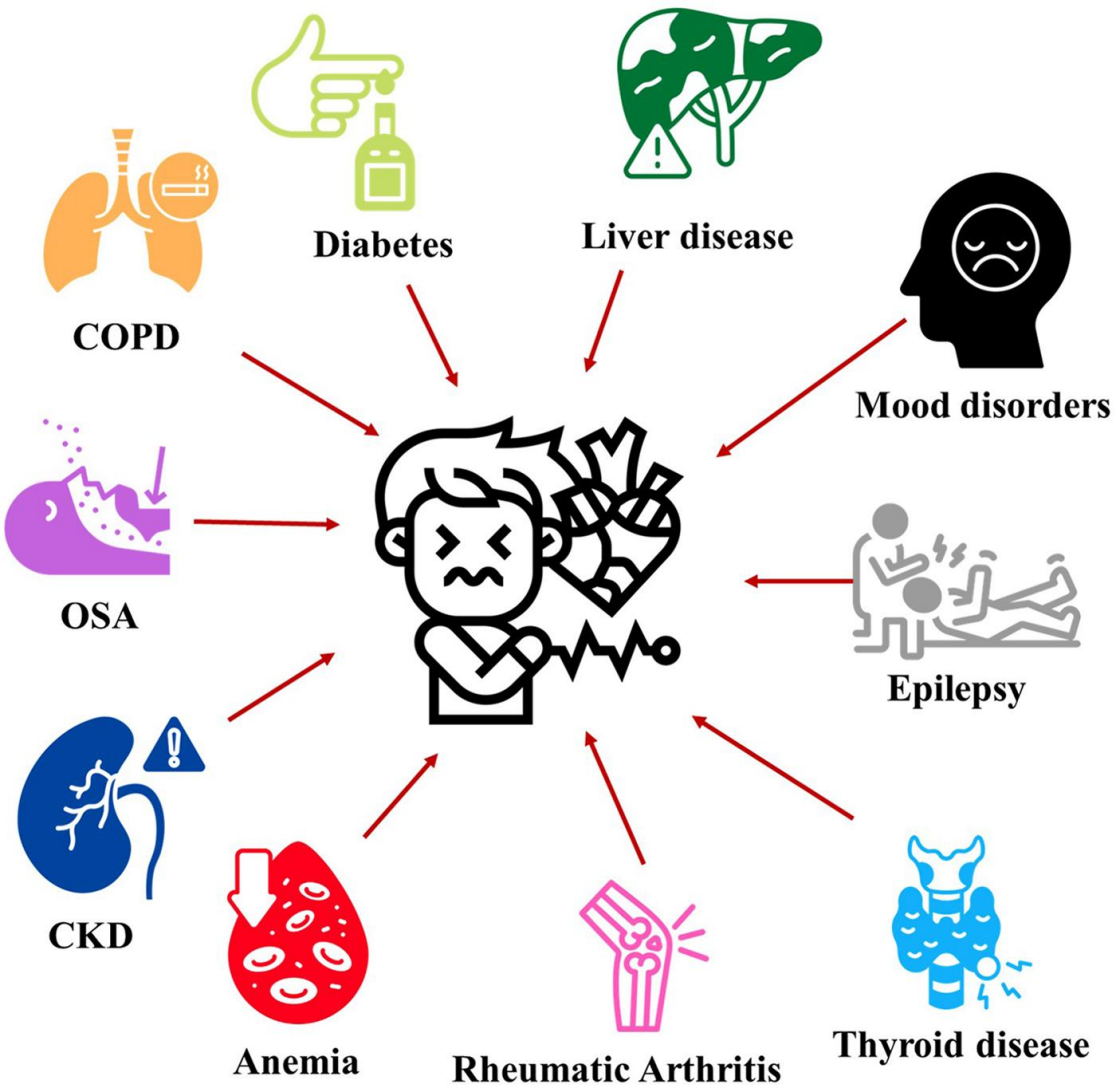
Contributive roles of non-cardiovascular comorbidities for sudden cardiac death. Non-cardiovascular comorbidities demonstrate significant associations with increased sudden cardiac death risk. Chronic kidney disease (CKD) confers the highest risk (HR 3.7, 95% CI 1.7-7.9), followed by type 1 diabetes (HR 2.4, 95% CI 2.0-3.0), elevated free T4 (HR 2.3 per 1 ng/dL increase, 95% CI 1.3-4.0), rheumatoid arthritis (RA; HR 1.9, 95% CI 1.1-3.6), epilepsy (HR 1.8, 95% CI 1.5-2.2), type 2 diabetes (HR 1.7, 95% CI 1.6-1.9), obstructive sleep apnea with apnea-hypopnea index >20 (OSA; HR 1.6, 95% CI 1.1-2.2), mood disorders (HR 1.6, 95% CI 1.4-1.9), metabolic dysfunction-associated steatotic liver disease (MASLD; HR 1.6, 95% CI 1.4-1.7), chronic obstructive pulmonary disease (COPD; HR 1.3, 95% CI 1.1-1.7), and anemia (HR 1.2 per 1 g/dL hemoglobin decrease in males, 95% CI 1.1-1.3; HR 1.2 in females, 95% CI 1.1-1.4). HR, hazard ratio; OR, odds ratio.

Predicting SCD is challenging due to several factors. First, although the definition of SCD was defined clearly as unexpected death due to a likely cardiac etiology, occurring rapidly after symptom onset when witnessed or if unwitnessed, within 24 h of the subject being last seen in their usual state of health (9), various existing studies implemented different definitions and methods, using national level data based on death certificates or International Classification of Diseases code such as CDC WONDER to detailed in house adjudication using information from Emergency Medical Service reports, medical records, and autopsy findings (10–14). This inconsistency makes evaluating and predicting SCD difficult. Second, approximately 50% of SCD cases, the cardiac arrest is the first manifestation of heart disease, with warning signs that range from classic symptoms like chest pain and shortness of breath to non-specific ones such as nausea, seizure-like episodes, and fatigue, which can be subtle and difficult to recognize, thus complicating risk prediction efforts (15, 16). Most SCD cases occur within the general population, who often displays none or only a few risk factors. This contrasts with a smaller group of individuals with cardiovascular disease (CVD) who have a higher risk and incidence of SCD but represent a lower absolute number (17, 18). Third, temporal variability adds another layer of complexity. SCD risk fluctuates across timescales ranging from circadian patterns and acute environmental exposures (such as heatwaves) to longitudinal changes in individual risk profiles through development of new comorbidities, progression of existing conditions, or therapeutic intervention, with aging serving as a powerful independent predictor (19, 20). This dynamic nature has not been adequately studied, adding to the complexity of risk assessment. Finally, and perhaps most critically, traditional cardiovascular comorbidities—coronary artery disease, congestive heart failure, atrial fibrillation, syncope, and stroke—have been extensively documented and incorporated into predictive risk scores. However, non-cardiovascular comorbidities remain insufficiently evaluated and underutilized in clinical algorithms, despite evidence suggesting they contribute to nearly 40% of SCD cases through non-cardiac mechanisms (18). These non-cardiac factors not only influence traditional cardiovascular conditions and worsen outcomes in acute decompensation but can also serve as primary triggers for arrest through pathways that remain poorly understood. Previous investigations have demonstrated the predictive value of non-cardiac comorbidities, both for overall SCD risk stratification and for distinguishing initial cardiac rhythms—a differentiation with profound implications for implantable cardioverter-defibrillator (ICD) placement in primary prevention, given that device benefit is limited to shockable rhythms (ventricular fibrillation/ventricular tachycardia) (Figure 1). This narrative review examines the evidence from studies using all-cause SCD as the primary outcome, focusing on non-cardiac comorbidities—an essential yet underrecognized factor in SCD prediction that demands increased clinical and research focus.

## 2 Predictive value of non-cardiac comorbidities for SCD

### 2.1 Neurologic conditions

Epilepsy affects over 51 million people worldwide, carrying a mortality rate 2–3 times higher than the general population, largely attributable to sudden unexpected death in epilepsy (SUDEP) (21, 22). A previous cohort study of more than 26,000 participants from Taiwan National Health Insurance Research Database demonstrated that patients with epilepsy had 1.8-fold [Hazard Ratio (HR) 1.8, 95% CI 1.5–2.2] increased risk of SCD compared to those without epilepsy during up to 17 years of follow-up (23). A case-control study utilized data from a large scale general practitioner research database with 926 SCD cases and 9,832 controls further revealed that the severity of SCD risk in patients with epilepsy also depends on whether the epilepsy is symptomatic [Odd Ratio (OR) 5.8, 95% CI 2.1–15.6] or stable (OR 1.6, 95% CI 0.7–4.1), the presence of seizure episode triggers, and particularly the use of antiepileptic medications (AEM), which can potentially trigger SCD/SUDEP in certain situations (24). Sodium channel-blocking AEM particularly increase vulnerability (OR 2.8, 95% CI 1.1–7.2), with carbamazepine and gabapentin showing the strongest association (OR 3.2 and 5.7, respectively) (24). Moreover, patients with epilepsy are less likely to have witnessed cardiac arrests or receive bystander CPR, which in part contributes to their significantly lower survival rate compared to non-epileptic subjects (25, 26). Cardiac conduction dysfunction with increased QTc, calcium channel dysfunction, autonomic dysfunction with decreased heart rate variability (HRV), and hypoxemia due to respiratory distress during seizure episodes along with catecholamine surge are potential predisposing and peri-ictal factors that induce SCD/SUDEP in this population (27–29).

Mood disorders represent another critical neuropsychiatric link to SCD. Depression amplifies SCD risk by 1.6-fold (HR 1.6, 95% CI 1.4–1.9) according to a systematic review and meta-analysis of four studies with SCD as the primary outcome, encompassing over 83,000 patients (30). This association is particularly pronounced in the elderly, with a population-based study from Northern Finland demonstrating a 2.7-fold increased risk (HR 2.7, 95% CI 1.4–5.5) in individuals ≥70 years old over an 8-year follow-up period (31). Meanwhile, a cohort study of 1,012 ICD patients demonstrated that individuals with high anxiety levels based on the State-Trait Anxiety Inventory had a 1.9-fold increased risk of ventricular arrhythmia during the first year following ICD implantation compared to those with low anxiety levels (32). The anxiety-SCD association appears particularly prominent in women, as evidenced by a cohort study utilizing the Nurses' Health Study database. Among 72,359 women without baseline history of cardiovascular disease or cancer, those with phobic anxiety (Crown-Crisp index score ≥4) exhibited a 1.6-fold increased risk of SCD (33). Autonomic dysregulation is the key mechanism linking mood disorders to SCD through increased sympathetic and decreased parasympathetic activity, leading to reduced heart rate variability, and increased ventricular arrhythmia (31, 32). Moreover, a population-based study from Denmark reported that antidepressant use is a risk factor for SCD, with risk escalating from 1.6 times for short-term exposure (1–5 years) to 2.2 times for prolonged (6+ years) (34). Some types of antidepressants, particularly tricyclic antidepressants (TCA), have been associated with increased risk of SCD when the dose is high (≥300 mg of amitriptyline or its equivalent), with a 2.5 times increased risk (95% CI, 1.04–6.12) (35). TCAs have been proven to have significant dose-related adverse effects on the cardiovascular system, such as orthostatic hypotension, tachycardia, decreased heart rate variability, and QT prolongation.

### 2.2 Respiratory conditions

Regarding respiratory diseases, chronic obstructive pulmonary disease (COPD) and obstructive sleep apnea (OSA) emerge as prominent predictors. A population-based study utilizing the Rotterdam Study database (approximately 15,000 participants aged ≥45 years with up to 24 years of follow-up) found that COPD was associated with an increased risk of SCD (HR 1.3, 95% CI 1.1–1.7). Notably, the risk escalated to 3.6-fold (HR 3.6, 95% CI 2.4–5.4) among COPD patients with frequent exacerbations (defined as ≥2 hospitalizations for moderate or severe exacerbations per year) (36). These findings were confirmed by a community-based case-control study in Oregon, USA [the Oregon Sudden Unexpected Death Study (ORSUDS) with catchment population approximately 1 million], which demonstrated a significant association between COPD and SCD (OR 2.2, 95% CI 1.4–3.5) after multivariable adjustment (37). In patients with COPD, chronic hypoxemia depletes cellular energy reserves, predisposing to non-shockable rhythms where cardiomyocytes cannot sustain adequate function, resulting in electromechanical dissociation or pump failure (38). Type 3 pulmonary hypertension drives right ventricular remodeling with increased arrhythmic susceptibility (39), while persistent systemic inflammation—mediated by pulmonary-derived cytokines—directly triggers ventricular dysrhythmias (40).

On the other hand, OSA presents a distinct pathophysiology characterized by repetitive nocturnal hypoxia-arousal cycles and intermittent autonomic activation. In a Minnesota cohort study by Gami et al. involving 10,701 adults who underwent polysomnography with follow-up from 1987 to 2003, OSA severity markers were significant predictors of SCD. Specifically, an apnea-hypopnea index (AHI) >20 conferred an HR of 1.6, while mean nocturnal O₂ saturation <93% was associated with an HR of 2.9 (41). This pattern generates oxidative stress, ventricular remodeling from supply-demand mismatch, and ectopic activity culminating in potentially fatal arrhythmias (42, 43). Additionally, a *post hoc* analysis of 112 SCD cases from the study above revealed an important finding: patients with severe OSA (AHI ≥40) were significantly more likely to experience cardiac arrest during sleep between midnight and 6 a.m. compared to those without OSA [Relative Risk (RR) 2.6, 95% CI 1.3–5.4]. This nocturnal timing pattern reduces the likelihood of witnessed events and access to bystander resuscitation, thereby further increasing SCD risk (44).

### 2.3 Gastrointestinal disorders

Hepatic pathology constitutes the primary gastrointestinal link to SCD. A recent large-scale study of approximately 5.4 million adults aged 20–39 years from the Korean National Health Insurance Service database (2009–2012, followed through 2020) investigated metabolic dysfunction-associated steatotic liver disease (MASLD) as a predictor of sudden cardiac arrest (SCA). MASLD was determined by the fatty liver index (FLI), and participants with high FLI (≥60) demonstrated a 1.6-fold increased risk of SCA (HR 1.6, 95% CI 1.4–1.7) (45). While the exact mechanisms remain poorly understood, SLD potentially facilitates SCD through metabolic syndrome-induced non-ischemic cardiomyopathy and chronic low-grade inflammation promoting arrhythmogenic ventricular fibrosis (46). Supporting this hypothesis, previous studies have documented significant ECG changes in liver disease patients. One study comparing 94 cirrhosis patients with 37 controls with mild chronic active hepatitis found prolonged QTc intervals (440.3 ± 3.2 ms vs. 393.6 ± 3.7 ms) (47), while another demonstrated QTc prolongation in 69 patients with alcoholic liver disease compared to age- and sex-matched healthy non-drinking controls (450 ms vs. 439 ms) (48). Furthermore, autopsy analysis from an ongoing community-based study in Southern California (2015–2023) [PREdiction of Sudden Death in Multi-ethnic Communities (PRESTO)] of 162 SCD cases revealed that SCD cases with SLD exhibited higher body mass index, greater hepatic mass, and increased alcohol consumption rates, yet paradoxically demonstrated lower coronary artery disease prevalence (49% vs. 71%) compared to those without SLD (49). These findings underscore the importance of non-cardiovascular mechanisms in SCD pathogenesis.

### 2.4 Kidney disease

Renal dysfunction progressively amplifies SCD risk from moderate impairment through end-stage kidney disease (ESKD). A study of 15,792 middle-aged individuals (aged 45–64 years at baseline) from the community-based Atherosclerosis Risk in Communities (ARIC) cohort demonstrated that advanced chronic kidney disease (CKD stage 3b or worse) was independently associated with SCD risk (HR 3.7, 95% CI 1.7–7.9) compared to individuals with eGFR ≥90 ml/min per 1.73 m^2^ (50). This association was further elaborated in a recent case-control study utilizing the ORSUDS cohort for discovery and the PRESTO study for validation. In ORSUDS, which included 2,068 SCA cases and 852 controls without history of ventricular arrhythmia or SCA, moderate CKD (stages 3a and 3b) emerged as an independent risk factor for SCA (OR 1.3, 95% CI 1.0–1.7). Notably, SCA risk increased by 24% for every 10 ml/min per 1.73 m^2^ decrease in eGFR below 90 ml/min per 1.73 m^2^ (51). These findings were validated in the PRESTO study including 817 SCA cases and 3,249 controls, which confirmed that moderate CKD was independently associated with SCA (OR 1.5, 95% CI 1.2–2.0), with a 41% increased risk for every 10 ml/min per 1.73 m^2^ decrease in eGFR below 90 ml/min per 1.73 m^2^. CKD might induce SCD through neurohormonal activation (sympathetic nervous system and renin–angiotensin–aldosterone system hyperactivity), systemic inflammation, volume overload, and electrolyte disorders (52, 53). These pathophysiological disturbances trigger myocardial hypertrophy, fibrosis, and adverse ventricular remodeling while simultaneously inducing both macrovascular atherosclerosis and microvascular arteriolosclerosis, impairing coronary perfusion. The resultant ischemic injury not only precipitates heart failure but also creates an arrhythmogenic substrate characterized by heterogeneous scar tissue that facilitates reentrant circuits and conduction system abnormalities, heightening susceptibility to fatal ventricular arrhythmias (52, 54). Particularly, in patients with ESKD undergoing dialysis, SCD contributes to a large portion of cause of death ranging from 14% to 45% with an overall incidence rate from 4.5 to 7 per 100,000 hemodialysis sessions (55–58). The hemodialysis procedure itself may represent an independent risk factor for SCD. A study by Bleyer et al. analyzing data from five dialysis centers in the southeastern U.S. (1995–2003) demonstrated a 1.7-fold increased risk of SCD during the 12-hour period following dialysis initiation compared to expected mortality rates (59). This temporal relationship was further investigated by a study of 32,065 participants in the End-Stage Renal Disease Clinical Performance Measures Project, a nationally representative sample of U.S. hemodialysis patients. Over 2.2 years of follow-up, the SCD rate following a long interdialytic interval (Mondays and Tuesdays, after the weekend gap) was significantly higher than on other days of the week (60). Similar findings were confirmed in a recent ORSUDS analysis, which reported a significantly higher proportion of SCA cases on dialysis days (nearly 3-fold higher than expected by random distribution), particularly following long interdialytic periods (Mondays and Tuesdays) (61). Although the complete mechanistic pathway from dialysis to SCD requires further elucidation, current literature has identified several arrhythmogenic contributors. Significant variation of intravascular volume, uremic toxic accumulation, coupled with derangements in serum electrolyte concentrations and their subsequent rapid normalization (62, 63).

### 2.5 Hematologic disorder

Anemia independently predicts SCA in the general population, as demonstrated by a Korean National Health Insurance Database Cohort study of approximately 500,000 individuals with a mean follow-up of 5.4 years. This study reported that SCA risk increased progressively across anemia severity categories, with HRs ranging from 1.5 to 6.2 in men and 1.9 to 8.8 in women from mild to severe anemia, respectively (64). Furthermore, SCD risk increased by 21%–24% per 1 g/dl decrease in hemoglobin, with inverse correlations observed between hemoglobin levels and ECG abnormalities such as QTc prolongation. A subsequent study utilizing the Mayo Clinic Aortic Stenosis database (8,357 adults with severe aortic stenosis) with SCD ascertainment through the National Death Index and medical records confirmed that anemia history was associated with significantly increased SCD risk (HR 1.4, 95% CI 1.1–1.9) (65). The main mechanistic consequence of anemia is reduced of oxygen content in blood which subsequently induces or worsens the myocardial ischemia causing increasing compensatory sympathetic signal such as tachycardiac and cardiac contraction. This phenomenon can cause cardiac remodeling with increasing of ventricular fibrosis, hypertrophy, and also electrophysiological alteration (66, 67).

### 2.6 Endocrine disorders

Diabetes consistently appears as an independent risk factor for SCD across multiple studies. A systematic review and meta-analysis of 19 population-based and 10 patient-based prospective studies encompassing over 6,000 SCD cases from more than 300,000 participants demonstrated a summary RR of 2.0 (95% CI 1.8–2.3) for SCD in individuals with diabetes compared to those without diabetes (68). This association was confirmed in two large Danish nationwide studies using death certificate-defined SCD from 2000 to 2010. The first study focused on younger individuals (aged 1–49 years) and reported incidence ratios of 8.6 (95% CI 5.8–12.8) in the 1–35 age group and 6.1 (95% CI 4.7–7.8) in the 36–49 age group for SCD in those with diabetes compared to non-diabetic persons (69). The second, more recent study investigated the entire Danish population in 2010 (approximately 5.5 million individuals) and revealed differential effects by diabetes type: type 1 diabetes conferred an HR of 2.4 (95% CI 2.0–3.0) for SCD, while type 2 diabetes showed an HR of 1.7 (95% CI 1.6–1.9) (70). This differential risk between diabetes types was previously reported by a community-based case-control study using the ORSUDS database, which included 2,771 SCA cases and 8,313 controls matched by sex, age, race/ethnicity, and geography. This analysis showed that the odds of SCA were 2.4-fold higher in type 1 diabetes compared to type 2 diabetes (71). Evidence is also accumulating regarding prediabetes and SCD risk. The aforementioned meta-analysis reported an RR of 1.2 (95% CI 1.1–1.4) for SCD in prediabetic individuals (68). Additionally, a population-based study in Eastern Finland of 2,641 men aged 42–60 years with 19 years of follow-up demonstrated that nondiabetic men with impaired fasting plasma glucose had a 1.5-fold increased risk of SCD (RR 1.5, 95% CI 1.1–2.1) compared to those with normal fasting glucose levels (72). Diabetes can contribute to SCD both by causing coronary artery disease, and by acting as a risk factor through various mechanisms. These include abnormal myocardial metabolism due to impaired insulin signaling, autonomic nervous system dysfunction as a diabetic complication, increased ventricular fibrosis leading to structural remodeling, and the risk of hypoglycemia (73).

An analysis from the Rotterdam Study, a prospective population-based cohort, including 10,318 participants with a median follow-up of 9.2 years, revealed that elevated free T4 levels were associated with increased SCD risk (HR 2.3 per 1 ng/dl increase, 95% CI 1.3–4.0) (74). Additionally, a similar population-based cohort study in Finland (5,211 participants aged ≥30 years, enrolled 2000–2001, median follow-up 13.2 years) demonstrated that elevated baseline TSH levels were also associated with higher SCD risk (HR 2.3, 95% CI 1.1–4.6) compared to TSH within the normal range (75). Thyroid hormones are known to increase heart rate, conduction velocity, and myocardial contractility by increasing the number of beta-adrenergic receptors, thereby heightening susceptibility to arrhythmias (76, 77).

### 2.7 Immunologic diseases

While immunologic mechanisms are well-established contributors to SCD risk through direct cardiovascular involvement in systemic conditions—particularly myocarditis, cardiac sarcoidosis, cardiac amyloidosis, systemic lupus erythematosus, and systemic sclerosis—this review focuses on non-cardiovascular immune-mediated conditions such as rheumatoid arthritis (RA), for which evidence remains limited. The association between RA and SCD was demonstrated in a population-based cohort study from Rochester, Minnesota, which included 603 residents diagnosed with RA using the American College of Rheumatology 1987 criteria and 603 age- and sex-matched non-RA controls. Beginning 2 years after RA diagnosis, patients with RA had a 1.9-fold increased risk of SCD (95% CI 1.1–3.6) compared to those without RA (78). The pathogenic mechanisms likely involve persistent high-grade systemic inflammation with elevated cytokine levels, which may induce cardiac fibrosis and autonomic cardiac dysfunction. Supporting this hypothesis, 76% of RA patients demonstrated prolonged corrected QT interval at baseline, which rapidly normalized following anticytokine therapy with tocilizumab in a case series of 17 RA patients receiving long-term treatment (79).

### 2.8 Non-cardiovascular comorbidities as predictors of shockable vs. non-shockable rhythms

Predicting SCD risk represents only the initial step in prevention efforts. Equally critical is identifying which patients will present with shockable vs. non-shockable rhythms, as our primary preventive device – the ICD – provides benefit exclusively for shockable presentations. However, the current indicator for ICD implantation, left ventricular ejection fraction (LVEF), demonstrates poor performance in predicting SCD, particularly among patients with prior myocardial infarction (MI) with c-statistic ranging from 0.50 to 0.56, as reported by a pooled analysis of 20 datasets encompassing more than 140,000 post-MI patients (80). Non-cardiovascular comorbidities may substantially improve our ability to predict initial cardiac arrest rhythm and identify appropriate ICD candidates.

Emerging evidence from community-based research reveals those non-cardiovascular comorbidities significantly drive arrest patterns toward non-shockable rhythms (asystole or pulseless electrical activity – PEA) (81, 82). Specifically, Neurological disorders confer 1.6-fold elevated risk for non-shockable presentations (95% CI 1.4–1.9) (82). Epilepsy patients demonstrate particularly striking differences, with 74% presenting in non-shockable rhythms compared to 56% of controls (*p* < 0.05) (26). Depression shows similarly pronounced effects: 73.2% vs. 51.2% non-shockable presentations (*p* < 0.05), translating to an odds ratio of 2.7 (95% CI 2.5–3.0) (83–85). Psychotropic medications—including antidepressants, antipsychotics, and anxiolytics—further amplify this vulnerability (82). COPD severity correlates directly with non-shockable risk, escalating from mild disease (OR 1.5, 95% CI 1.3–1.7) to severe obstruction (OR 2.0, 95% CI 1.8–2.2) (82, 86). Hepatic pathology demonstrates comparable patterns: cirrhotic patients present with non-shockable rhythms in 75% of cases vs. 42% without cirrhosis (*p* < 0.001) (87, 88). CKD associates with predominantly non-shockable presentations across multiple community studies (51, 89). Particularly, Implantable loop recorder data from hemodialysis patients reveal bradycardia and asystole—rather than ventricular arrhythmias—as the dominant terminal rhythms (90). Machine learning analyses additionally identify anemia as a predictor of pulseless electrical activity (89). Finally, Diabetes increases non-shockable risk (OR 1.3, 95% CI 1.2–1.4), with type 1 diabetes potentially conferring greater vulnerability than type 2 (71, 82). While diverse pathways may explain these associations, a shared mechanism centers on cardiomyocyte energy depletion leading to electromechanical dissociation. Hypoxemia (from anemia, COPD, seizure-related respiratory distress), uremic toxicity (CKD), and impaired glucose utilization (diabetes) converge on cellular energy failure—a state where electrical activity persists despite insufficient contractile function to generate effective cardiac output.

## 3 Risk prediction models incorporating non-cardiovascular comorbidities

Multiple risk prediction models for SCD have developed across diverse populations, from community-based cohorts to individuals with established cardiovascular disease. Predictive algorithms generally draw from four domains: patient demographics, comorbidity profiles, diagnostic test findings (including ECG, echocardiography, CT, and CMR), and genetic characteristics. Among these models, those incorporating non-cardiovascular comorbidities as predictive components are detailed in Table 1 (12, 91–99). The majority of predictive models originated from the United States (8) or Europe (2), demonstrating good to excellent discriminative ability (c-indices 0.68–0.82) with both internal and external validation. These algorithms incorporate diverse variables spanning demographic characteristics, clinical conditions, laboratory values, imaging findings, and genetic markers.

**Table 1:** Summarization of clinical studies incorporating non-cardiovascular comorbidities as risk predictors.

Authors (year)	Study design and population characteristics	Predictive component	Main results
Atwater et al. (2009) (12)	Duke Databank for Cardiovascular Disease: Adults ≥18 years undergoing cardiac catheterization (1/1985–5/2005) with ≥75% diameter stenosis in ≥1 native coronary artery	Reduced LVEF, number of diseased coronary arteries, DM, HTN, CHF, CVD, smoking	Duke SCD risk score: Internally validated via bootstrapping (c-index 0.75); externally validated in ischemic cardiomyopathy cohort from Sudden Cardiac Death Heart Failure Trial (c-index 0.64).
Deo et al. (2011) (91)	Heart and Estrogen/progestin Replacement Study: Postmenopausal women <80 years without prior hysterectomy, with history of MI, CABG, PCI, or >50% angiographic coronary stenosis	LVEF, >1 MI, CHF, eGFR <40 ml/min/1.73 m^2^, AF, physical activity, DM	The risk model demonstrated a c-index of 0.681 which is significantly better than LVEF alone (c-index 0.600) with a net improvement of 0.20 (*o* < 0.001).
Adabag et al. (2014) (92)	Irbesartan in Patients with Heart Failure and Preserved Ejection Fraction trial: Patients >60 yrs with LVEF ≥45%, NYHA class II-IV, and ≥1 CHF hospitalization within prior 6 months.	Age, Male, DM, MI, LBBB on ECG, NT-proBNP	The SCD prediction model showed a C-index of 0.75. The observed cumulative incidence of SCD in high-risk group were 11% versus 4% in low-risk group based on the SCD prediction model.
Kraaier et al. (2014) (93)	Academic Medical Center (Amsterdam) & Thorax Center Twente (Enschede): Patients with ischemic or dilated cardiomyopathy and LVEF ≤ 35%, receiving primary prevention ICD implantation (4/2002–12/2008).	Age > 75 years, AF, LVEF ≤20%, eGFR ≤30 ml/min/1.73 m^2^	Risk stratification: Low (≤1 factor), intermediate (2 factors), high (≥3 factors) with 1-yr mortality of 3.4%, 10.9%, 38.9% (*p* < 0.01), respectively. External validation (Erasmus Medical Center, Rotterdam): 1-yr mortality 2.5%, 13.2%, 46.3% (*p* < 0.01) across risk groups.
Deo et al. (2017) (94)	From the Atherosclerosis Risk in Communities Study: Individuals from 45 to 64 years old at baseline, without established cardiovascular disease, and complete data.	Age, Male, African American race, current smoking, systolic blood pressure, use of antihypertensive medication, DM, serum potassium, serum albumin, HDL, eGFR, QTc interval	Over 10-years, the SCD risk score demonstrated an excellent discrimination (c-index of 0.82) in discovery and 0.75 validated cohorts of Cardiovascular Health Study.
Bogle et al. (2019) (95)	From the Atherosclerosis Risk in Communities Study: Individuals from 45 to 64 years old at baseline. Caucasian and African-American cohorts were analyzed separately.	Age, sex, total cholesterol, usage of lipid-lowering and antihypertensive medication, blood pressure, smoking, DM, BMI	The risk score demonstrated good internal discrimination with c-indices of 0.82 for white and 0.75 for black populations. External validation in the Framingham Heart Study showed a c-index of 0.82.
Adabag et al. (2020) (96)	Treatment of Preserved Cardiac Function Heart Failure with an Aldosterone Antagonist Trial: Patients with symptomatic heart failure and LVEF ≥45% and either CHF hospitalization within prior 12 months or BNP ≥100 pg/mL or NT-proBNP ≥360 pg/mL	Age, sex, MI, DM, LBBB on ECG, NT-pro BNP	Patients with high-risk of SCD classified by the risk score had 3.7-fold higher risk of SCD than low-risk. The SCD risk model showed a c-index of 0.74.
Ohlsson et al. (2020) (97)	The Malmö Diet and Cancer Study: Women aged 44–73 yrs and men aged 46–73 years enrolled 1991–1996. Study data were subsequently merged with the local cardiac arrest registry maintained at SUS University Hospital in Malmö.	GRS, Age, Male, Smoking, DM, HTN, high ApoB	The novel composite risk score including risk factors and GRS predicted sudden cardiac arrest with a HR = 110.81 (95% CI, 15.43–795.63) for the highest (5) versus the lowest quintile (1) of the risk score.
Chugh et al. (2022) (98)	Oregon Sudden Unexpected Death Study: Subjects age ≥18 with lifetime clinical history available from archived medical records were selected.	DM, MI, AF, CVD, CHF, COPD, Seizure, Syncope, Heart rate >75, prolonged QTc, Tpe >89 ms, delayed intrinsicoid deflection ≥50 ms, LVH by echocardiogram	The VFRisk score showed very good discrimination power with c-index of 0.808 and internally validated with c-index of 0.776. The risk score also validated externally with c-index 0.782 which overperformed LVEF ≤35% with c-index of 0.638.
Truyen et al. (2025) (99)	Framingham Heart Study, two cohorts were included: the Original cohort of 5,209 individuals aged 28–62 years at baseline, and the Offspring cohort of 5,124 participants consisting of Original cohort members’ children and their spouses.	DM, MI, AF, CVD, CHF, COPD, Seizure, Syncope, Heart rate >75, Tpe >89 ms, prolonged QTc, LVH by echocardiogram	The VFRisk score was validated with good discrimination with the c-index of 0.71. A 1-unit increase in VFRisk score was associated with a 48% increase in odds of SCD. The highest VFRisk quartile had 7.8-fold higher odds of SCD than the lowest quartile

LVEF, left ventricular ejection fraction; DM, diabetes mellitus; HTN, hypertension; CHF, congestive heart failure; CVD, cerebrovascular Disease; SCD, sudden cardiac death; eGFR, estimated glomerular filtration rate; AF, atrial fibrillation; MI, myocardial infarction; CABG, coronary artery bypass graft; PCI, percutaneous coronary intervention; NYHA, New York Heart Association; LBBB, left bundle branch block; QTc, corrected QT interval; Tpe, Tpeak-tend; LVH, left ventricular hypertrophy; COPD, chronic obstructive pulmonary disease; GRS, genetic risk score.

### 3.1 Non-cardiovascular comorbidities in current models and the paradox of underutilization

Among non-cardiac conditions, only diabetes, CKD (or eGFR-estimated renal function), COPD, and seizure disorders appear in existing algorithms. Diabetes emerges as the most frequently included, featuring in 7 of 9 models—highlighting its critical pathophysiologic role in SCD. The VFRisk score, developed by Chugh et al. from the ORSUDS and PRESTO (subsequently validated by Truyen et al. in the Framingham Heart Study cohort), stands alone as the only algorithm incorporating three non-cardiac conditions (diabetes, COPD, seizure disorder) (98, 99). Notably, VFRisk was also uniquely designed to differentiate shockable from non-shockable initial rhythms. Recent machine learning approaches have further advanced rhythm prediction, distinguishing PEA from ventricular fibrillation using novel predictors including anemia, CKD, OSA, cancer, and mood disorders (89). Despite abundant evidence linking non-cardiovascular conditions to SCD, their limited incorporation into clinical prediction tools remains concerning. Several factors explain this disconnect:

### 3.2 Historical focus and knowledge gaps

The cardiac-centric approach reflects long-established associations between cardiovascular conditions and SCD, whereas non-cardiac relationships emerged more recently with incompletely understood mechanisms. Many foundational studies excluded these variables from initial design, making them unavailable for multivariable analysis—particularly in *post-hoc* analysis. Cardiologists' relative unfamiliarity with non-cardiac conditions may also contribute to their omission from study protocols.

### 3.3 Sample size constraints

SCD low incidence creates significant statistical challenges. Traditional multivariable logistic regression requires 10–20 events per variable, meaning 15 covariates demand 150–300 SCD cases—beyond most institutions' capacity. Additionally, clinical utility demands simplicity; overly complex algorithms fail adoption in daily practice. These pragmatic constraints prioritize established cardiac factors over emerging non-traditional predictors.

### 3.4 Complex disease interactions

The complicated relationships between non-cardiac and cardiovascular conditions remain poorly characterized, with overlapping effects potentially diluting individual contributions after multivariable adjustment. For instance, CKD achieved statistical significance in VFRisk univariable analysis but lost predictive value in the multivariable model (98). The combinatorial complexity of comprehensively modeling all potentially relevant cardiac and non-cardiac conditions creates prohibitive statistical burdens unfeasible for most investigations.

### 3.5 Overfitting risk and generalizability

Incorporating numerous covariates substantially increases overfitting risk, compromising model reproducibility across populations and limiting external validation success. This technical limitation creates systematic bias favoring traditional, well-validated cardiac factors over newer non-cardiac predictors that require larger datasets to demonstrate stable, generalizable effects.

## 4 The future of risk prediction model for SCD

Acknowledging the inherent difficulty of predicting and preventing SCD, we believe future studies should aim not only to predict SCD events but also to identify the presenting initial rhythm, thereby optimizing patient selection for ICD implantation. Specifically, for individuals identified as high-risk for SCD without ICD indication, more comprehensive and real-time monitoring strategies could be implemented leveraging advances in wearable devices with integrated artificial intelligence. Real-time monitoring may enable detection or even short-term prediction of SCD, potentially improving resuscitation response times, increasing witnessed event rates, and ultimately enhancing cardiac arrest survival while reducing SCD mortality. Non-cardiac conditions are expected to play a crucial role in these future risk models. While substantial challenges remain, we propose the following strategies as potential solutions:

### 4.1 Multidisciplinary research collaboration

Cross-specialty partnerships in study design and analysis can address inherent limitations of cardiac-centric approaches. Engaging neurologists, nephrologists, pulmonologists, psychiatrists, and endocrinologists provides essential expertise for accurately measuring systemic diseases and interpreting their complex interactions with cardiovascular pathophysiology. Such collaboration reduces specialty bias while enhancing mechanistic understanding of how non-cardiovascular conditions contribute to SCD risk.

### 4.2 Multicenter collaboration and data pooling

Large-scale collaborative networks substantially increase SCD event number, overcoming sample size constraints of single-center investigations. Pooling data across institutions can achieve thousands of SCD cases, enabling robust multivariable models that accommodate numerous predictors. This expanded statistical capacity creates room for both traditional cardiac factors and emerging non-cardiac variables without compromising model stability.

### 4.3 Advanced analytical approaches in the machine learning era

Supervised ML using tree-based algorithms (random forests, gradient boosting) automatically identify non-linear relationships and complex interactions without requiring prior specification. Feature importance metrics systematically rank predictors, enabling development of parsimonious models that retain crucial non-cardiac variables while maintaining clinical practicality. This approach balances comprehensiveness with the simplicity demanded for bedside implementation, potentially enhancing discrimination in large datasets with intricate patterns. Moreover, clustering techniques—including K-means, hierarchical clustering, and model-based methods—uncover hidden patient patterns and novel phenotypes. These approaches can reveal how specific combinations of cardiac and systemic conditions create distinct risk profiles, generating hypotheses about disease synergy that traditional regression frameworks cannot detect. Such insights deepen mechanistic understanding beyond additive risk factor models. Advanced regularization techniques (LASSO, elastic net) and dimensionality reduction methods (principal component analysis) might address collinearity among clustered conditions—hypertension-diabetes-kidney disease or depression-anxiety-cardiac arrest—that confound conventional regression. These methods extract meaningful risk signals from correlated predictor sets, resolving a fundamental challenge in multimorbidity modeling. ML capacity to handle high-dimensional data, capture non-linear relationships, and identify complex interaction patterns positions it as a transformative tool for SCD risk stratification. However, realizing this potential requires rigorous external validation, attention to overfitting prevention, and preservation of clinical interpretability. Success ultimately depends not only on statistical sophistication but also on meaningful collaboration between data scientists, clinicians, and implementation specialists to bridge the gap between algorithmic performance and bedside utility.
